# Associations Between Neighborhood Disinvestment and Breast Cancer Outcomes Within a Populous State Registry

**DOI:** 10.1002/cncr.33900

**Published:** 2021-09-08

**Authors:** Jesse J. Plascak, Andrew G. Rundle, Xinyi Xu, Stephen J. Mooney, Mario Schootman, Bo Lu, Jason Roy, Antoinette M. Stroup, Adana A. M. Llanos

**Affiliations:** 1 Comprehensive Cancer Center, The Ohio State University, Columbus, Ohio; 2 Division of Cancer Prevention and Control, Department of Internal Medicine, College of Medicine, The Ohio State University, Columbus, Ohio; 3 Department of Epidemiology, Mailman School of Public Health, Columbia University, New York, New York; 4 Department of Statistics, College of Arts and Sciences, Columbus, Ohio; 5 Department of Epidemiology, School of Public Health, University of Washington, Seattle, Washington; 6 Department of Clinical Analytics, SSM Health, St Louis, Missouri; 7 Division of Biostatistics, College of Public Health, Columbus, Ohio; 8 Department of Biostatistics and Epidemiology, School of Public Health, Piscataway, New Jersey; 9 Rutgers Cancer Institute of New Jersey, New Brunswick, New Jersey; 10 New Jersey State Cancer Registry, New Jersey Department of Health, Trenton, New Jersey

**Keywords:** breast cancer outcomes, built-environment physical disorder, cancer registry linkage, neighborhood disinvestment

## Abstract

**BACKGROUND:**

Breast cancer (BrCa) outcomes vary by social environmental factors, but the role of built-environment factors is understudied. The authors investigated associations between environmental physical disorder—indicators of residential disrepair and disinvestment—and BrCa tumor prognostic factors (stage at diagnosis, tumor grade, triple-negative [negative for estrogen receptor, progesterone receptor, and HER2 receptor] BrCa) and survival within a large state cancer registry linkage.

**METHODS:**

Data on sociodemographic, tumor, and vital status were derived from adult women who had invasive BrCa diagnosed from 2008 to 2017 ascertained from the New Jersey State Cancer Registry. Physical disorder was assessed through virtual neighborhood audits of 23,276 locations across New Jersey, and a personalized measure for the residential address of each woman with BrCa was estimated using universal kriging. Continuous covariates were z scored (mean ± standard deviation [SD], 0 ± 1) to reduce collinearity. Logistic regression models of tumor factors and accelerated failure time models of survival time to BrCa-specific death were built to investigate associations with physical disorder adjusted for covariates (with follow-up through 2019).

**RESULTS:**

There were 3637 BrCa-specific deaths among 40,963 women with a median follow-up of 5.3 years. In adjusted models, a 1-SD increase in physical disorder was associated with higher odds of late-stage BrCa (odds ratio, 1.09; 95% confidence interval, 1.02–1.15). Physical disorder was not associated with tumor grade or triple-negative tumors. A 1-SD increase in physical disorder was associated with a 10.5% shorter survival time (95% confidence interval, 6.1%−14.6%) only among women who had early stage BrCa.

**CONCLUSIONS:**

Physical disorder is associated with worse tumor prognostic factors and survival among women who have BrCa diagnosed at an early stage.

## INTRODUCTION

Breast cancer (BrCa) morbidity and mortality vary substantially by numerous social and built-environmental factors within the United States.^1–3^ For example, US women residing in areas of lower socioeconomic composition are more likely to be diagnosed with breast tumors characterized by a poorer prognosis—metastatic stage, higher grade (ie, poorly differentiated or undifferentiated tumor cells), and triple-negative BrCa (TNBC) subtype (negative for estrogen receptor, progesterone receptor, and HER2 receptor)—and experience poorer BrCa survival compared with women living in higher socioeconomic composition areas.^1–4^ Despite associations with indicators of BrCa morbidity and mortality, measures of residential socioeconomic composition are nonspecific or overlap with individual socioeconomic measures.^5^ Moreover, study of the residential socioeconomic environment is limited by the challenges of intervening on such factors.^6^ Converging bodies of literature suggest that additional social and built-environmental factors might constitute specific and feasible targets to reduce BrCa morbidity and mortality.^7–9^

Physical disorder is a modifiable indicator of disinvestment, amenable to practice and policy interventions (eg, vacant lot remediation, neighborhood revitalization),^10,11^ that might be associated with BrCa outcomes through various pathways, including health behaviors or psychosocial stress.^7^ Visual indicators of physical disorder (eg, the presence of garbage, graffiti, or abandoned buildings; building conditions in disrepair; etc) have been associated with known and probable risk factors of aggressive BrCa tumor phenotypes and survival, including obesity, alcohol use, tobacco smoking, DNA methylation, and perceived stress.^12–15^ Institutional racism has resulted in the colocation of neighborhoods of higher physical disorder, lower socioeconomic composition, and larger racial/ethnic minority populations,^16,17^ suggesting that physical disorder might also influence socioeconomic and racial/ethnic disparities in BrCa outcomes.

Despite recommendations to integrate cancer epidemiologic data sets with observed built-environment factors (eg, sidewalk walkability, physical disorder),^7,18^ only 1 recent study has investigated any such relation with cancer-related outcomes.^19^ The study among 215 African American (AA) patients with BrCa enrolled in a longitudinal behavioral intervention trial in St Louis, Missouri, yielded unexpected associations: residence near lower quality sidewalks was associated with greater improvements in emotional well-being reported over time compared with women living near higher quality sidewalks. There was a lack of evidence for associations between the presence of garbage/graffiti or abandoned buildings and other patient-reported quality-of-life outcomes, indicating that larger studies are needed. There are also no studies of relations between physical disorder and BrCa clinical outcomes, including no studies of pathways to survival involving clinicopathologic features (eg, disease stage, tumor grade, and tumor subtype).

Recent technologic and methodologic advancements have permitted large-scale visual assessments and address-level estimates of physical disorder-related characteristics within the state of New Jersey, which reports all cancer cases diagnosed among residents to the high-quality New Jersey State Cancer Registry (NJSCR).^17,20^ The objective of the current study was to test associations between physical disorder and BrCa stage, grade, subtype, and survival among women diagnosed with invasive BrCa while residing in New Jersey.

## MATERIALS AND METHODS

### Study Sample and Data Collection

Data were abstracted from the NJSCR for all New Jersey female residents aged 20 years and older who were diagnosed with BrCa between 2008 and 2017 with their first primary, histologically confirmed, invasive breast tumor (n = 57,173). Because physical disorder is typically considered an urban construct,^21^ data were restricted to the most urban census tracts (Rural-Urban Commuting Area = *metropolitan area core*), which encompassed 93% of the New Jersey general population and BrCa cases (n = 53,369) that meet the above eligibility criteria.^22^ Sociodemographic characteristics (age, race, ethnicity, primary payer health insurance, geocoded residential address, date of diagnosis), tumor clinicopathologic characteristics (stage at diagnosis, grade, subtype), and vital status (cause and date of death) were obtained from NJSCR records. Race and ethnicity—conceptualized as intrapersonal (ie, self-identifying) and interpersonal (others labeling the race or ethnicity of others based mainly on visual appearance) products of racism (for details, see Supporting Figs. 1 and 2) based on *ecosocial theory*^23^—were combined into non-Latina White, non-Latina Black, non-Latina Asian/Pacific Islander/Native American/other, and Latina in regression analyses, and subcategories were analyzed within exploratory analyses. In this framework, persistent and large disparities of BrCa clinicopathologic features and survival are primarily influenced by racism, both historical and current. Health insurance type was collapsed into private, uninsured, Medicaid, Medicare, and other (for details, see Supporting Materials). Stage at diagnosis was determined according to Surveillance, Epidemiology, and End Results Collaborative Stage 2000, and any missing or unknown values were set according to Surveillance, Epidemiology, and End Results Summary Stage 2000. Stage was dichotomized into early (localized and regional) and late (distant). Tumor grade was dichotomized into low (well differentiated and moderately well differentiated) and high (poorly differentiated and undifferentiated). Subtype was collapsed into TNBC versus non-TNBC. BrCa-specific death was based on International Classification of Diseases, tenth revision codes C50 through C50.9.

**Figure 1.**
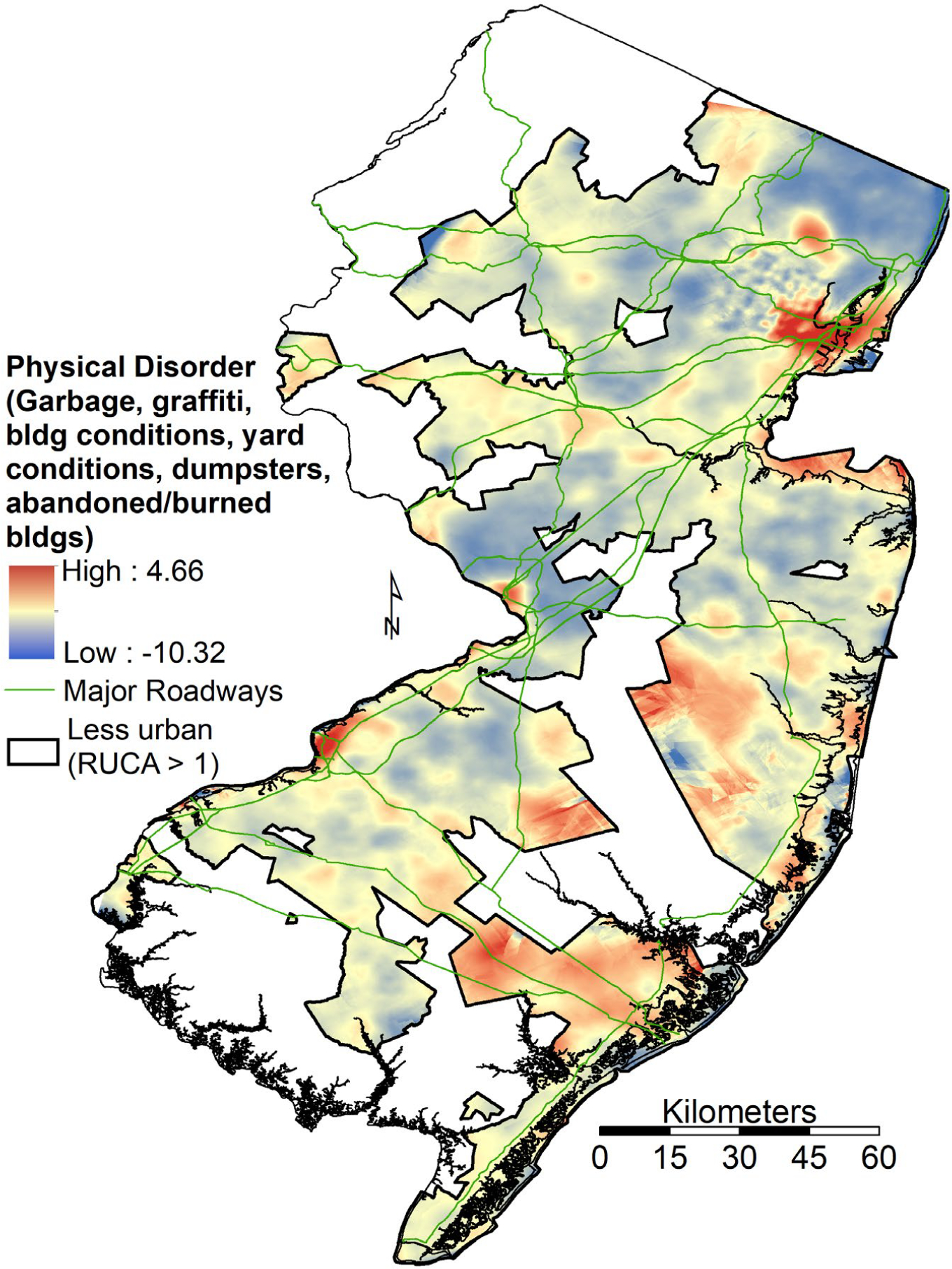
Residential physical disorder is illustrated among New Jersey urban regions. Bldg indicates building; RUCA, Rural-Urban Commuting Area.

Virtual neighborhood auditing, a method used to assess visual residential characteristics, of Google Street View (GSV) scenes was conducted between January 2018 and June 2019 at 23,276 urban point locations across New Jersey.^20,21^ Residential audit locations were randomly selected along nonhighway roads and were independent of BrCa case residential locations. The auditing platform CANVAS was used to assess 6 physical disorder-related characteristics with previously verified measurement properties: garbage/litter (yes/no), graffiti (yes/no), boarded up or burned out buildings (yes/no), large dumpsters (none, 1–2, >2), building conditions (very good, moderate, fair, poor), and yard conditions (very good, moderate, fair, poor).^17,20,21^ Test-retest assessments by 4 trained auditors following a standardized protocol resulted in at least *substantial* reliability (κ ≥ 0.61) for all 9 items.^20^ A single factor representing physical disorder was created from nonmissing (n = 14,671; 63.0%) neighborhood audit item response patterns using item response theory.^21^ Internal consistency reliability of physical disorder was 0.965 using methods described by Thissen.^24^ Continuous surfaces of physical disorder values were estimated from a universal kriging spatial-prediction model of item response theory factor scores (Fig. 1) (for details, see Supporting Materials).^25^ Residential physical disorder was attributed to each BrCa case by kriging spatial predictions at their geocoded residential address at the time of diagnosis. The median GSV image date was October 2013 (10th to 90th percentile, August 2012 to September 2017).

Selected census-based, area-level covariates were calculated at the census tract level from 2010 decennial census data.^26^ African American (AA) and Latino residential segregation measures were estimated with the Gini and isolation indices using census block-level demographic data.^27^ The Gini index is a common measure of segregation *evenness*, and the isolation index is a measure of *exposure*.^27^ Similar to previous studies, we calculated proportions of AA (% AA) and Latino (% Latino) populations as measures of racial/ethnic density.^2,3^ Neighborhood socioeconomic composition was based on vigintiles of the Yost index as previously linked to cancer registries.^28^ Population density was calculated as the population per square kilometer.^26^ Primary care physician density (per 100,000 population) was ascertained from the Robert Wood Johnson County Health Ranking and was available at the county level.^29^***Statistical Analysis***

The total analytic sample after excluding unknown and missing values for tumor subtype (n = 6055; 11.3%), tumor grade (n = 5444; 10.2%), health insurance status (n = 2866; 5.4%), disease stage (n = 1642; 3.1%), socioeconomic composition (n = 1087; 2.0%), follow-up time (n = 446; 0.8%), geocoded address (n = 55; 0.1%), AA segregation measures (n = 31; <0.1%), and Latino segregation measures (n = 5; <0.1%) was 40,963. Sociodemographic, tumor, and neighborhood covariates were summarized as means or frequencies and as standard deviations (SDs) or percentages by levels of residential physical disorder (high/low median split). Continuous covariates were z scored (mean-centered, standardized by dividing by the SD) to reduce collinearity in models. Logistic regression models of late-stage BrCa, high-grade BrCa, and TNBC were created to calculate odds ratios (ORs) and 95% confidence intervals (CIs) by physical disorder unadjusted for other covariates (model 1) and adjusted for age, race/ethnicity, health insurance, diagnosis year, other tumor prognostic factors, and area-level covariates (model 2). Accelerated failure time (AFT) models were built to estimate survival time to BrCa-specific death by levels of physical disorder. AFT models are more appropriate than Cox proportional hazard models for mediation analysis.^30,31^ Patients who did not experience BrCa-specific mortality were right censored at the date of mortality from other causes or on December 31, 2018. Time ratios (TRs) and 95% CIs were calculated from 3 models: 1) residential physical disorder alone, 2) model 1 plus potential confounders (age, race/ethnicity, health insurance, diagnosis year, area-level covariates), and 3) model 2 plus tumor prognostic factors (tumor stage, grade, and subtype). For interpretability, we presented TR results as percent changes in survival time (100% × [TR − 1]). We explored causal mediation of the physical disorder-BrCa survival relation by each tumor prognostic factor under the following conditions: 1) physical disorder was associated with the tumor prognostic factor in covariate-adjusted logistic regression models, and 2) physical disorder was associated with survival time in confounder-adjusted AFT models (model 2).^30–32^ The mediator models were based on the conceptual framework and causal graph depicted in the Supporting Materials. Natural indirect effects and the proportions mediated were calculated from 1000 bootstraps. We tested multiplicative interactions only between physical disorder and tumor prognostic factors that met the criteria for mediation testing. We conducted 3 sensitivity analyses: 1) missing data imputation in which AFT model 3 was recalculated 8 times based on the 8 possible combinations of imputed values for dichotomous tumor stage, grade, and subtype; 2) limiting neighborhood audit data to 2007 through 2013 GSV images (n = 8718) and limiting NJSCR cases to those diagnosed during 2014 through 2017 (n = 18,057) to investigate the robustness of results to reverse direction of associations; and 3) Cox proportional hazard shared frailty (census tract clustering) models. Analyses were conducted between June and July 2020 using SAS version 9.4 and ArcGIS version 10.6. This study protocol was approved by a local institutional review board.

## RESULTS

Regions of high physical disorder (Fig. 1, red hues) are found in the Northeast border (eg, Newark, Jersey City, Union City), Southwest border (eg, Trenton and Camden), Southeast coastal areas (eg, Toms River, Atlantic City), and Southeast interior (eg, Hamilton Township) of New Jersey (Fig. 1). The average ± SD physical disorder value among cases was 0.06 ± 0.49 (minimum, −3.42; maximum, 2.48).

Distributions of sociodemographic, tumor, and area-level factors by median physical disorder are shown in Table 1 (also see Supporting Table 1). Greater than 69% of non-Latina Black and Latina women resided at addresses characterized as high physical disorder compared with 43% of non-Latina White women. Physical disorder was also higher among those who were uninsured or had Medicaid, were diagnosed at a late stage, had high-grade tumors, had a TNBC subtype, or resided in areas with lower socioeconomic composition and primary care physician density and areas with higher AA and Latino density, AA and Latino segregation (isolation index), and population density.

After adjusting for covariates, the odds of late-stage BrCa at diagnosis increased to 1.08 (95% CI, 1.02–1.15) for a 1-SD increase in physical disorder (Table 2, model 2; for full model results, see Supporting Table 2). The adjusted estimated odds of a high-grade tumor or TNBC according to changes in physical disorder were close to 1.0 and had wide confidence intervals.

The median follow-up was 5.3 years (95% CI, 5.3–5.4 years), and there were 3639 BrCa-specific deaths. The estimated 5-year survival rate was 91.1% (95% CI, 90.8%−91.4%). In models adjusted for potential confounders, each 1-SD increase in residential physical disorder was associated with −8.6% (95% CI, −12.9%, −4.0%) shorter survival time (Table 3, model 2; for full model results, see Supporting Table 3). In models that included tumor clinicopathologic factors, the relation between physical disorder and survival time depended on stage at diagnosis; increases in physical disorder were associated with shorter survival time only among women who had early stage BrCa at diagnosis (Table 3, model 3).

Mediation analysis indicated a very small, natural, indirect effect involving physical disorder, stage at diagnosis, and survival time. The natural indirect effect of the associations between physical disorder and survival time according to stage at diagnosis was −0.34% (95% CI, −0.07%, −0.65%). The association between physical disorder and survival measured by the natural direct effect was −10.4% (95% CI, −5.8%, −14.8%). This corresponded to only 2.9% of the association between physical disorder and survival time mediated by tumor stage.

Sensitivity analyses based on a Cox proportional hazard, shared frailty model (see Supporting Table 4) as well as sensitivity using imputed combinations of stage, grade, and TNBC yielded results that were qualitatively unchanged from the main analyses. Sensitivity analyses limiting GSV images to those dated from 2007 to 2013 and limiting the years of BrCa diagnosis to 2014 through 2017 resulted in an estimated association between physical disorder and survival time in the final model (Table 3, model 3) that was attenuated toward the null (those with early stage BrCa: −6.5%; 95% CI, −13.5%, 1.0%; those with late-stage BrCa: −0.1%; 95% CI, −8.5%, 9.1%).

## DISCUSSION

By using novel data and methods, we investigated associations between visually observed, built-environment physical disorder linked to BrCa outcomes within a populous state cancer registry. Women residing at addresses with more visible indicators of physical disorder—the presence of garbage, graffiti, dumpsters, abandoned buildings, and poorer building and yard conditions—had greater odds of late-stage diagnosis compared with women residing at addresses with less physical disorder. Similarly, greater physical disorder was associated with shorter BrCa-specific survival, but only among those diagnosed at an early stage. Results were qualitatively similar among imputations of missing disease stage, tumor grade, and TNBC subtype. Data restrictions ensuring that built-environment measurements occurred before BrCa diagnoses yielded attenuated associations, which could be caused by more accurate associations or reductions in sample size and less precise estimates. Together, the results of this study suggest that potential relations between BrCa survival and physical disorder may be restricted to early stage diagnoses and independent of tumor grade and subtype.

Observed physical disorder is considered an indicator of public and private disinvestment and has been associated with risk factors of BrCa outcomes, including greater alcohol consumption and tobacco use, lower physical activity, obesity, and perceived stress.^12,15,33^ Therefore, observed associations between physical disorder and BrCa survival could involve psychosocial and physiologic pathways. For example, genomic factors have been identified that could affect timing of and stage at BrCa diagnosis, including early onset BrCa and the development of more aggressive tumors.^34^ Cytokine products of the NOD-like receptor protein (NLRP) inflammasome pathway characterized from the tumor microenvironment have been associated with breast tumor progression.^35^ A study of neighborhood factors and peripheral blood DNA methylation among participants in a cardiovascular disease cohort found that a worse neighborhood social environment—based on a composite measure that included physical disorder indicators—was associated with increased *NLRP12* methylation and decreased gene expression.^36^ Thus a potential explanation for stage-dependent associations could reflect the gradual, but adversely accumulating, stressors through which long-term residence in areas of greater physical disorder might shorten BrCa survival time. Indeed, women with late-stage diagnoses had only 7% of the survival time that women with early stage diagnoses experienced, and this relatively short time may not allow for the accumulation of health-adverse physical disorder effects, such as alcohol or tobacco use, physical inactivity, or psychosocial stress and inflammation.

Only 1 known cancer-related study involving observed physical disorder found no evidence of associations between longitudinal quality-of-life outcomes and the presence of garbage/graffiti or abandoned buildings/lots among newly diagnosed AA BrCa survivors.^19^ That study used comparable virtual neighborhood audit data sources and methods but was limited by a small sample size of 215 women. Because of correlations with other covariates and anticipated small effect sizes, studies of built-environment factors require large sample sizes like the registry-based data set used for the current study.^15,17^

Strengths of this study include the novel application of verified methods, allowing the characterization of built-environment exposures for a large sample,^17,20^ and linkage to a high-quality, population-based cancer registry within the sociodemographically heterogeneous state of New Jersey.^26^ Numerous recommendations have been made for large-scale data integration of cancer outcome data with emerging data technologies.^7,18,37^ The NJSCR is a high-quality registry recognized for high data completeness and has <12% missing or unknown values for tumor grade and BrCa subtype. Moreover, imputation sensitivity analyses indicated no qualitative changes in BrCa survival results, reducing the likelihood of bias because of differential missing data.

The results of this study are potentially limited by several factors, including: unmeasured confounding, built-environment exposure misclassification, and lack of residential histories and longitudinal physical disorder measurement. Historical and current discriminatory practices and policies within real estate, mortgage lending, and home-owner/renter discrimination, as well as resources shared across racialized social networks in New Jersey, could confound the association between residential physical disorder and BrCa outcomes.^16^ Additional factors that were unmeasured in this study (eg, BrCa screening availability/concordance, health care access, treatment availability/concordance, diet, physical activity, socioeconomic factors, etc) could help clarify associations involving physical disorder. The GSV image dates on which built-environment characteristics were assessed may have changed over time and may not reflect the built environment at the time of diagnosis; in addition, women might have moved before or after diagnosis, such that built-environment characteristics of the address at diagnosis may misclassify levels of exposure. A recent study of BrCa survivors found that 22% moved their address over a 2-year period postdiagnosis.^19^ Longitudinal changes in built-environment characteristics of individuals have been understudied, but 1 recent residential history study of patients with colon cancer in New Jersey reported that only a small percentage of individuals had moved to neighborhoods of appreciably different poverty levels over a mean follow-up of 5.5 years.^38^ Future studies should include the measurement of longitudinal built-environment characteristics along with individual residential history information to allow for more accurate built-environment exposure calculation, investigation of time-varying associations, and exposure windows across the life course.

### Conclusion

Results indicating an association between greater physical disorder and shorter survival time among women with early stage (but not late-stage) diagnoses focuses attention on future studies of the exposure time-relevant mechanisms that may be responsible for such relations. Physical disorder is a novel characteristic of the built environment that is modifiable through community actions and local policies and is associated with BrCa outcomes, deserving further investigation.

## Supplementary Material

Supplement

## Figures and Tables

**TABLE 1.** Distribution of Sociodemographic, Tumor, and Area-Level Factors by Median Levels of Residential Physical Disorder, N = 40,963: New Jersey State Cancer Registry Breast Cancer Cases, 2008 to 2017

	Residential Physical Disorder: No. (%)	
Variable	Low: ≤Median	High: >Median
Age: Mean ± SD, y	60.2 ± 13.5	60.2 ± 13.7
Race ethnicity		
Non-Latina White	16,565 (57.0)	12,515 (43.0)
Non-Latina Black	1169 (23.9)	3720 (76.1)
Non-Latina Asian/Pacific	1707 (62.3)	1034 (37.7)
Islander, AI/AN, other		
Non-Latina Asian/Pacific Islander	1606 (63.5)	922 (36.5)
Non-Latina AI/AN	12 (37.5)	20 (62.5)
Non-Latina other	89 (49.2)	92 (50.8)
Latina	1305 (30.7)	2948 (69.3)
Mexican	50 (31.4)	109 (68.6)
Puerto Rican	152 (20.4)	593 (79.6)
Cuban	70 (30.2)	162 (60.8)
Dominican	52 (18.9)	223 (81.1)
Other	981 (34.5)	1861 (65.5)
Primary payer/health insurance status		
Private	11,158 (54.3)	9392 (45.7)
Uninsured	489 (35.3)	898 (64.7)
Medicaid	487 (26.5)	1353 (73.5)
Medicare	7207 (50.1)	7166 (49.9)
Other	1405 (49.9)	1408 (50.1)
Year of diagnosis		
<2013	9505 (51.0)	9121 (49.0)
≥2013	11,241 (50.3)	11,096 (49.7)
Cancer stage		
Early	19,799 (51.0)	19,032 (49.0)
Late	947 (44.4)	1185 (55.6)
Tumor grade		
Low	13,665 (51.9)	12,656 (48.1)
High	7081 (48.4)	7561 (51.6)
Tumor subtype		
Nontriple negative	18,685 (51.4)	17,641 (48.6)
Triple negative	2061 (44.4)	2576 (55.6)
Census-based neighborhood factors		
Socioeconomic composition: Mean ± SD, vigintile	16.7 ± 3.4	11.5 ± 5.2
AA residential density, %	5.4 ± 8.3	17.2 ± 23.3
AA residential segregation, Gini index^a^	69.8 ± 14.9	63.9 ± 15.7
AA residential segregation, isolation index^b^	12.8 ± 11.2	25.0 ± 23.2
Latino residential density, %	8.9 ± 8.7	19.8 ± 20.8
Latino residential segregation, Gini index^a^	52.3 ± 12.7	52.3 ± 15.1
Latino residential segregation, isolation index^b^	15.2 ± 10.3	26.6 ± 20.2
Population density per km^2^	1423 ±1547	3361 ± 4545
PCP density per 100,000 population	123.9 ± 36.3	100.5 ± 33.9

Abbreviations: AA, African American; AI, American Indian; AN, Alaska Native; PCP, primary care physician; SD, standard deviation; triple negative, negative for estrogen receptor, progesterone receptor, and HER2 receptor.

a The Gini index is a common measure of segregation *evenness,* and is scored on a scale from 0 to 100.

b The isolation index is a measure of *exposure* and is scored on a scale from 0 to 100.

**TABLE 2.** Odds Ratios of Late-Stage, High-Grade, and Triple-Negative Breast Cancer by Residential Physical Disorder, New Jersey State Cancer Registry Breast Cancer Cases, 2008 to 2017

	OR (95% CI) Associated With a 1-SD Change in Physical Disorder^a,b^		
Model	Late Stage	High Grade	TNBC
Model 1	1.18 (1.13–1.23)	1.08 (1.06–1.10)	1.19(1.15–1.22)
Model 2	1.08 (1.02–1.15)	0.97 (0.94–1.01)	1.01 (0.96–1.06)

Abbreviations: CI, confidence interval; OR, odds ratio; SD, standard deviation; TNBC, triple-negative breast cancer (negative for estrogen receptor, progesterone receptor, and HER2 receptor).

a The analysis was from separate logistic regression models of the probability of each outcome (late stage vs early stage, high-grade vs low-grade tumor, TNBC vs non-TNBC) and either was unadjusted for other covariates (model 1) or was adjusted for all covariates listed in Table 1, model 2 (for the full model 2, see Supporting Table 2).

b A 1-SD change in physical disorder = 0.49.

**TABLE 3.** Estimated Percent Changes in Survival Time to Breast Cancer Mortality by Residential Physical Disorder: New Jersey State Cancer Registry Breast Cancer Cases, 2008 to 2017

	% Change in Survival Time (95% CI)^a^		
Variable	Model 1^b^	Model 2^c^	Model 3^d^
Residential physical disorder, 1-SD change	−23.2 (−25.8, −20.5)	−8.6 (−12.9, −4.0)	Stage interaction
Among early stage			−10.5 (−14.6, −6.1)
Among late stage			−0.9 (−6.7, 5.3)

Abbreviations: CI, confidence interval; SD, standard deviation.

a Values are expressed as the percent change in survival time (time ratio − 1) × 100%; negative values indicate shorter survival time, and positive values indicate longer survival time.

b This model includes physical disorder only.

c This model includes model 1 plus age, race/ethnicity, health insurance status, year of diagnosis, socioeconomic composition, African American (AA) density, AA Gini segregation, AA isolation segregation, Latino density, Latino Gini segregation, Latino isolation segregation, population density, and primary care physician density.

d This model includes model 2 plus grade, subtype, and stage × physical disorder interaction (for full model results, see Supporting Table 3).
