# Supplementary material for: Associations Between Neighborhood Disinvestment and Breast Cancer Outcomes Within a Populous State Registry
Source: Cancer. Author manuscript; Available in PMC 2022 May 5. (PMC9070603; doi:10.1002/cncr.33900)
Supplement: Supplement [file NIHMS1798572-supplement-Supplement.docx]

**SUPPORTING MATERIALS**

**Materials and Methods**

*Race-ethnicity*

We use socio-ecologic theories of disease distribution, drawing heavily on *Ecosocial Theory¸*(Krieger, 2001; Krieger, 2011, 2013; Krieger, Jahn, & Waterman, 2017) to conceptualize race-ethnicity as an intra-(i.e., self-identifying) and inter-personal (others labeling the race-ethnicity of others based mainly on visual appearance) factor influenced by the U.S. context. In this framework, persistent and large disparities of breast cancer clinicopathologic features and survival are primarily influenced by racism, both historical and current. Historical structural racism (e.g., U.S. slavery of people form the African continent, forced treaties and land removal of First Nations/Native Americans, 20^th^ century housing covenants denying occupancy of people considered African American, mortgage lending discrimination in areas with a greater proportion of people considered non-White, etc.), especially, focuses attention on inequities that might operate over long spans of time and place to influence health behaviors/psychosocial factors (i.e., substance use, physical activity, diet, psychosocial stress, gene expression/phenotype), intra- and inter-personal social factors (socioeconomic status, interpersonal discrimination, social capital and social networks), in turn, effecting tumor initiation, progression, and outcomes, including breast cancer clinicopathologic features and survival (Supporting Figure 1) (Krieger, 2011; Saini et al., 2019; Williams, Mohammed, & Shields, 2016). Accordingly, adjustment for race-ethnicity could allow for a more accurate measure of association between physical disorder resulting from racism/disinvestment and breast cancer outcomes.

*Health Insurance*

Health insurance categories were created by collapsing levels of the primary payer at diagnosis variable (NAACCR item # 630): Private (‘Private Insurance: Managed care, HMO, or PPO’, ‘Private Insurance: Fee-for-Service’); Uninsured (‘Not insured’, ‘Not insured, self-pay’); Medicaid (‘Medicaid’, ‘Medicaid - Administered through a Managed Care plan’); Medicare (‘Medicare/Medicare, NOS’, ‘Medicare with supplement, NOS’, ‘Medicare - Administered through a Managed Care plan’, ‘Medicare with private supplement’, ‘Medicare with Medicaid eligibility’); Other (‘TRICARE’, ‘Military’, ‘Veterans Affairs’, ‘Indian/Public Health Service’, ‘Insurance, NOS’).

*Universal Kriging Method*

Continuous surfaces of physical disorder values were estimated from a Universal Kriging (UK) spatial prediction model based on IRT factor scores. The UK model was parameterized as follows: 3^rd^ order polynomial spatial de-trending prior to empirical semivariogram estimation, semivariogram binning of 100 lags each 200 meters apart (i.e., 20 km maximum distance), local Kriging with a minimum 40 and maximum of 120 observed IRT factor scores for each prediction. Leave-one-out cross-validation of predicted physical disorder resulted in a root-mean-square error (RMSE) of 0.646. Prediction accuracy was robust to UK model re-parameterization.

*Mediation Analyses*

Mediation analyses was guided by a conceptual framework (Supporting Figure 1) and directed acyclic graph (Supporting Figure 2) developed based on *Ecosocial Theory*.

Supporting Figure 1. Conceptual framework involving physical disorder, breast cancer tumor prognostic factors, and survival.


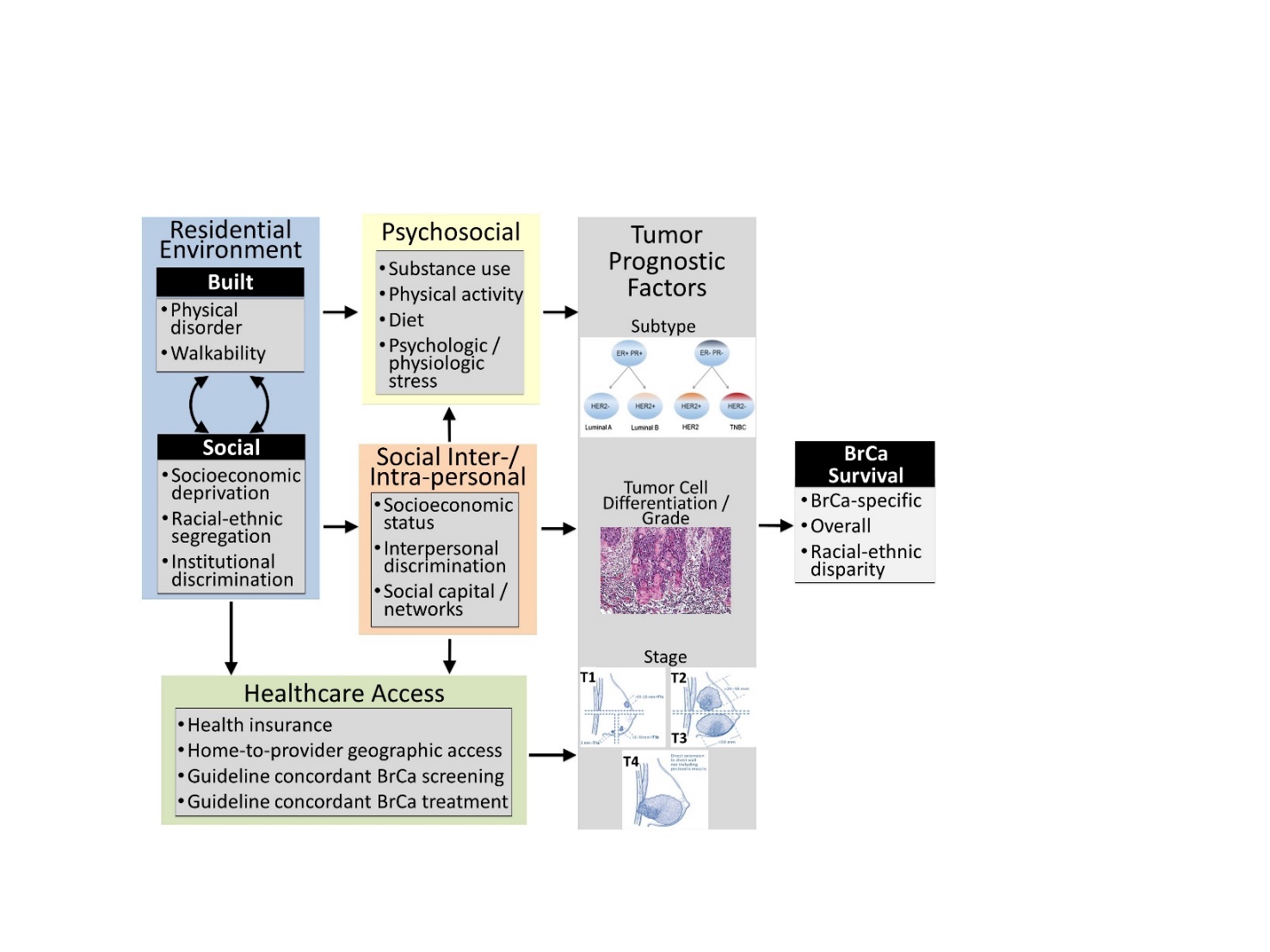


Supporting Figure 2. Directed acyclic graph depicting potential mediation models considered involving physical disorder and survival with mediation by stage at diagnosis, grade, and triple negative breast cancer^1^


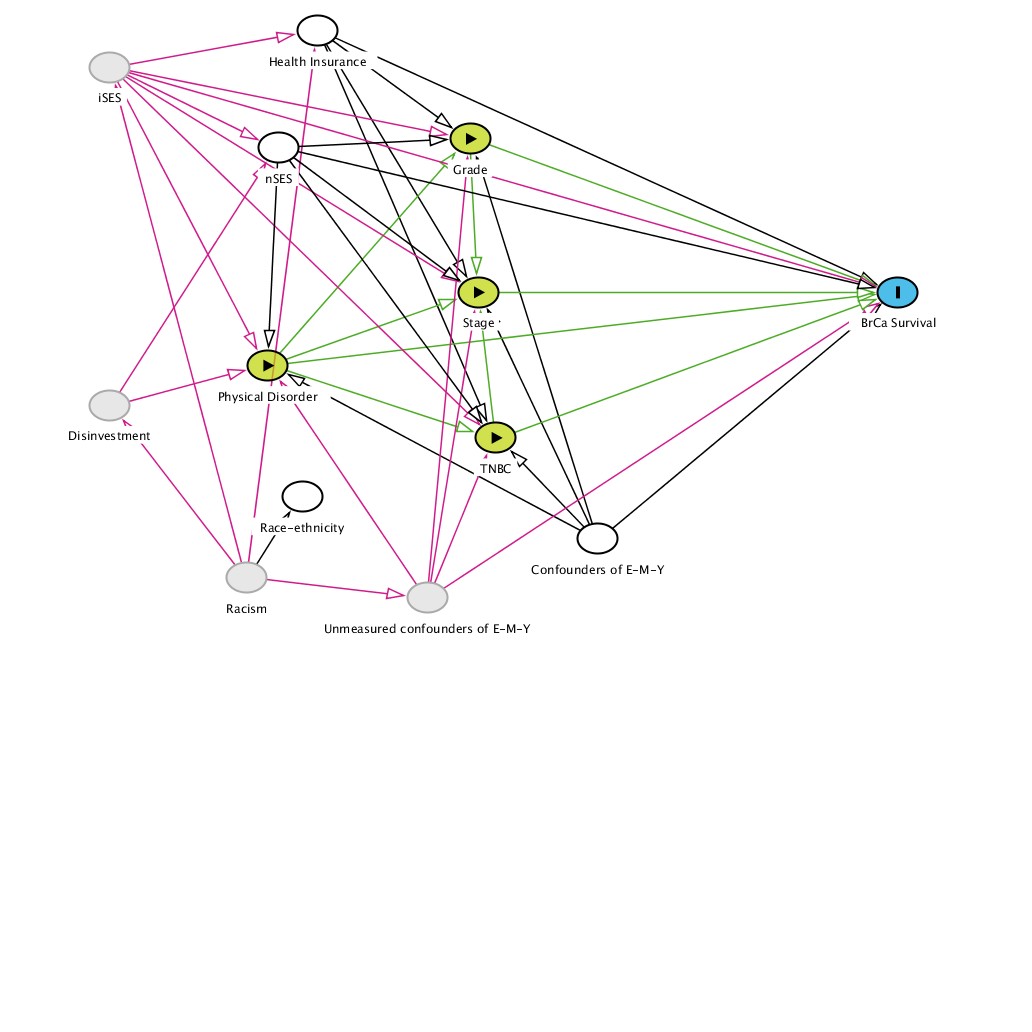


^1^ DAG was made using Daggity: Johannes Textor, Benito van der Zander, Mark S. Gilthorpe, Maciej Liskiewicz, and George TH Elli- ´ son. Robust causal inference using directed acyclic graphs: the R package ‘dagitty’. International Journal of Epidemiology, 45(6):1887–1894, December 2016

Daggity model code

dag {

bb="-2.618,-2.025,3.04,2.024"

"BrCa Survival" [outcome,pos="2.305,-0.294"]

"Confounders of E-M-Y" [adjusted,pos="0.659,1.163"]

"Health Insurance" [adjusted,pos="-0.880,-1.842"]

"Physical Disorder" [exposure,pos="-1.152,0.139"]

"Race-ethnicity" [adjusted,pos="-0.962,0.917"]

"Unmeasured confounders of E-M-Y" [latent,pos="-0.276,1.514"]

Disinvestment [latent,pos="-2.019,0.375"]

Grade [exposure,pos="-0.041,-1.203"]

Racism [latent,pos="-1.267,1.394"]

Stage [exposure,pos="0.003,-0.294"]

TNBC [exposure,pos="0.099,0.567"]

iSES [latent,pos="-2.019,-1.626"]

nSES [adjusted,pos="-1.092,-1.150"]

"Confounders of E-M-Y" -> "BrCa Survival"

"Confounders of E-M-Y" -> "Physical Disorder"

"Confounders of E-M-Y" -> Grade

"Confounders of E-M-Y" -> Stage

"Confounders of E-M-Y" -> TNBC

"Health Insurance" -> "BrCa Survival"

"Health Insurance" -> Grade

"Health Insurance" -> Stage

"Health Insurance" -> TNBC

"Physical Disorder" -> "BrCa Survival"

"Physical Disorder" -> Grade

"Physical Disorder" -> Stage

"Physical Disorder" -> TNBC

"Unmeasured confounders of E-M-Y" -> "BrCa Survival"

"Unmeasured confounders of E-M-Y" -> "Physical Disorder"

"Unmeasured confounders of E-M-Y" -> Grade

"Unmeasured confounders of E-M-Y" -> Stage

"Unmeasured confounders of E-M-Y" -> TNBC

Disinvestment -> "Physical Disorder"

Disinvestment -> nSES

Grade -> "BrCa Survival"

Grade -> Stage

Racism -> "Health Insurance"

Racism -> "Race-ethnicity"

Racism -> "Unmeasured confounders of E-M-Y"

Racism -> Disinvestment

Racism -> iSES

Stage -> "BrCa Survival"

TNBC -> "BrCa Survival"

TNBC -> Stage

iSES -> "BrCa Survival"

iSES -> "Health Insurance"

iSES -> "Physical Disorder"

iSES -> Grade

iSES -> Stage

iSES -> TNBC

iSES -> nSES

nSES -> "BrCa Survival"

nSES -> "Physical Disorder"

nSES -> Grade

nSES -> Stage

nSES -> TNBC

}

Mediation analyses were only tested for if physical disorder was associated with a tumor factor in logistic regression models adjusted for covariates (including the other tumor factor) and if physical disorder was associated with survival time in accelerated failure time models adjusted for covariates (excluding all of the three tumor factors depicted in Supporting Figure 2). As mediation analyses require interaction testing between the exposure and mediator, we also only tested for such interactions in instances where mediation was explored; the only survival time model in which an interaction was tested between physical disorder and stage.

The following equations defined the accelerated failure time outcome model, mediator logistic regression, natural direct, and natural indirect effects of mean breast cancer survival time on the ratio scale:

Mediator model,

*logit*$\left\{ P\left( M=1|A,C \right) \right\}$ = $\beta_{0}+\beta_{1}a+ \beta_{2}^{'}$*c*

outcome model,

$log$[$T$] *=* $\theta_{0}+{\theta_{1}a+\theta}_{2}m+\theta_{3}am+ \theta_{4}^{'}c+v\epsilon$

natural direct effect,

$E$[$T_{aM_{a^{*}}}$]/$E$[$T_{a^{*}M_{a^{*}}}$] *=* $\frac{\exp(\theta_{1}a)\left\{ 1+\exp\left( \theta_{2}+\theta_{3}a+\beta_{0}+\beta_{1}a^{*}+ \beta_{2}^{'}c \right) \right\}}{exp(\theta_{1}a^{*})\left\{ 1+\exp\left( \theta_{2}+\theta_{3}a^{*}+\beta_{0}+\beta_{1}a^{*}+ \beta_{2}^{'}c \right) \right\}}$

and natural indirect effect,

$E$[$T_{aM_{a}}$]/$E$[$T_{aM_{a^{*}}}$] *=* $\frac{\left\{ 1+\exp\left( \beta_{0}+\beta_{1}a^{*}+ \beta_{2}^{'}c \right) \right\}\left\{ 1+\exp\left( \theta_{2}+\theta_{3}a+\beta_{0}+\beta_{1}a+ \beta_{2}^{'}c \right) \right\}}{\left\{ 1+\exp\left( \beta_{0}+\beta_{1}a+ \beta_{2}^{'}c \right) \right\}\left\{ 1+\exp\left( \theta_{2}+\theta_{3}a+\beta_{0}+\beta_{1}a^{*}+ \beta_{2}^{'}c \right) \right\}}$

where $T$ is breast cancer-specific failure time, $M$ is the mediator stage at diagnosis,$A$ is the main exposure physical disorder, $C$ represents the covariates, $\beta$ represent the coefficients of relationships involving odds of stage at diagnosis (late vs early), *c* are the specific covariates included (see Methods), $m$ is late-stage at diagnosis, $\theta$ represent the coefficients of relationships involving breast cancer-specific survival time,$a$ is the baseline physical disorder exposure (here, the mean value), $\epsilon$ is residual error and follows the extreme value distribution, $v$ is the shape parameter following a Weibull distribution, $a$* is the change in physical disorder exposure (here, 1 standard deviation increase).

As sensitivity analysis to Table 1, physical disorder median and interquartile range values by levels of covariates were calculated.

**SUPPORTING RESULTS**

Supporting Table 1. Median and interquartile range values of residential physical disorder by levels of sociodemographic, tumor, and area-level factors, n=40,963 NJSCR BrCa cases 2008-2017

|  | **Physical disorder Median (IQR)** |
| --- | --- |
| Age, years |  |
| < 60 | 0.03 (-0.24 ̶ 0.29) |
| ≥ 60 | 0.04 (-0.22 ̶ 0.28) |
| Race-ethnicity |  |
| Non-Latina, White | -0.02 (-0.26 ̶ 0.18) |
| Non-Latina, Black | 0.37 (0.06 ̶ 0.90) |
| Non-Latina, Asian/Pacific Islander/AI/AN/Other^1^ | -0.09 (-0.37 ̶ 0.18) |
| Latina | 0.31 (-0.05 ̶ 0.67) |
| Primary payer/health insurance |  |
| Private | 0.00 (-0.27 ̶ 0.23) |
| Uninsured | 0.23 (-0.12 ̶ 0.62) |
| Medicaid | 0.32 (0.01 ̶ 0.77) |
| Medicare | 0.04 (-0.22 ̶ 0.28) |
| Other | 0.04 (-0.18 ̶ 0.33) |
| Year of diagnosis |  |
| < 2013 | 0.03 (-0.24 ̶ 0.28) |
| ≥ 2013 | 0.04 (-0.23 ̶ 0.29) |
| Cancer stage |  |
| Early | 0.03 (-0.24 ̶ 0.28) |
| Late | 0.08 (-0.17 ̶ 0.39) |
| Grade |  |
| Low | 0.02 (-0.24 ̶ 0.27) |
| High | 0.06 (-0.22 ̶ 0.32) |
| Tumor subtype |  |
| Non-triple negative | 0.03 (-0.24 ̶ 0.27) |
| Triple negative | 0.09 (-0.18 ̶ 0.38) |
| Census tract-level |  |
| Socioeconomic composition, vigintile |  |
| < 15 | 0.29 (0.04 ̶ 0.58) |
| ≥ 15 | -0.12 (-0.38 ̶ 0.07) |
| AA residential density, %^2^ |  |
| < 3.9 | -0.08 (-0.35 ̶ 0.14) |
| ≥ 3.9 | 0.15 (-0.10 ̶ 0.44) |
| AA residential segregation, Gini Index (0-100)^2^ |  |
| < 60.8 | 0.10 (-0.15 ̶ 0.41) |
| ≥ 60.8 | -0.04 (-0.33 ̶ 0.18) |
| AA residential segregation, Isolation Index (0-100)^2^ |  |
| < 11.1 | -0.07 (-0.32 ̶ 0.15) |
| ≥ 11.1 | 0.14 (-0.11 ̶ 0.40) |
| Latino residential density, % |  |
| < 7.4 | -0.06 (-0.30 ̶ 0.13) |
| ≥ 7.4 | 0.16 (-0.13 ̶ 0.46) |
| Latino residential segregation, Gini Index (0-100) |  |
| < 52.2 | 0.01 (-0.26 ̶ 0.29) |
| ≥ 52.2 | 0.06 (-0.20 ̶ 0.28) |
| Latino residential segregation, Isolation Index (0-100) |  |
| < 14.5 | -0.07 (-0.30 ̶ 0.12) |
| ≥ 14.5 | 0.17 (-0.13 ̶ 0.46) |
| Population density, per square kilometer |  |
| < 1196.7 | 0.16 (-0.04 ̶ 0.41) |
| ≥ 1196.7 | -0.12 (-0.43 ̶ 0.14) |
| Primary care physician density, per 100,000 people |  |
| < 114.0 | -0.04 (-0.28 ̶ 0.15) |
| ≥ 114.0 | 0.13 (-0.16 ̶ 0.46) |

^1^ AI = American Indian, AN = Alaska Native

^2^ AA = African American

Supporting Table 2. Adjusted odds ratios of late-stage, high grade, and TNBC by covariate levels, NJSCR BrCa cases, 2008-2017

|  | OR (95% CI) | | |
| --- | --- | --- | --- |
|  | Late-stage | High grade | TNBC |
| Age, 1 yr | 1.01 (1.01,1.02) | 0.98 (0.98,0.98) | 1.00 (0.99,1.00) |
| Race ethnicity |  |  |  |
| Non-Latina, White | 1.00 | 1.00 | 1.00 |
| Non-Latina, Black | 1.11 (0.94,1.30) | 1.49 (1.37,1.62) | 1.77 (1.58,1.98) |
| Non-Latina, Asian/Pacific Islander/AI/AN/Other^1^ | 0.74 (0.60,0.90) | 1.23 (1.13,1.34) | 0.91 (0.79,1.04) |
| Latina | 0.62 (0.52,0.74) | 1.13 (1.04,1.22) | 1.09 (0.97,1.24) |
| Primary payer/health insurance |  |  |  |
| Private | 1.00 | 1.00 | 1.00 |
| Uninsured | 3.02 (2.51,3.63) | 1.15 (1.02,1.30) | 1.15 (0.97,1.35) |
| Medicaid | 2.30 (1.92,2.75) | 1.15 (1.03,1.28) | 0.99 (0.85,1.15) |
| Medicare | 1.16 (1.01,1.33) | 0.99 (0.92,1.06) | 1.15 (1.04,1.28) |
| Other | 0.95 (0.78,1.17) | 0.87 (0.80,0.95) | 0.84 (0.73,0.97) |
| Year of diagnosis, per 1 standard deviation (sd) | 1.03 (0.98,1.07) | 0.91 (0.89,0.94) | 0.93 (0.90,0.96) |
| Cancer stage |  |  |  |
| Early |  | 1.00 | 1.00 |
| Late |  | 2.34 (2.13,2.57) | 0.89 (0.78,1.02) |
| Grade |  |  |  |
| Low | 1.00 |  | 1.00 |
| High | 2.34 (2.13,2.57) |  | 9.33 (8.63,10.08) |
| Tumor subtype |  |  |  |
| Non-triple negative | 1.00 | 1.00 |  |
| Triple negative | 0.89 (0.78,1.01) | 9.33 (8.64,10.08) |  |
| Census-based neighborhood factors, per 1 sd |  |  |  |
| Socioeconomic composition | 0.98 (0.92,1.05) | 1.00 (0.96,1.04) | 0.98 (0.93,1.03) |
| AA residential density^2^ | 0.93 (0.79,1.10) | 0.98 (0.90,1.07) | 1.03 (0.91,1.17) |
| AA residential segregation, Gini Index ^2^ | 1.03 (0.96,1.10) | 0.99 (0.96,1.02) | 1.00 (0.96,1.05) |
| AA residential segregation, Isolation Index ^2^ | 1.09 (0.94,1.27) | 1.02 (0.94,1.10) | 1.00 (0.89,1.12) |
| Latino residential density | 0.89 (0.71,1.14) | 0.96 (0.86,1.09) | 1.08 (0.91,1.29) |
| Latino residential segregation, Gini Index | 0.91 (0.85,0.98) | 0.99 (0.96,1.03) | 1.03 (0.98,1.09) |
| Latino residential segregation, Isolation Index | 1.09 (0.88,1.37) | 1.09 (0.97,1.22) | 0.94 (0.80,1.11) |
| Population density | 1.06 (1.00,1.13) | 1.06 (1.03,1.08) | 0.89 (0.86,0.93) |
| Primary care physician density | 0.95 (0.90,1.00) | 1.02 (0.99,1.06) | 1.00 (0.95,1.05) |

^1^ AI = American Indian, AN = Alaska Native

^2^ AA = African American

Supporting Table 3. Estimated percent changes in survival time of BrCa mortality by covariate levels, NJSCR BrCa cases, 2008-2017^1^

|  | TR (95% CI) |
| --- | --- |
| Age, 1 yr | -1.9 (-2.2,-1.6) |
| Race ethnicity |  |
| Non-Latina, White | Reference |
| Non-Latina, Black | -23.9 (-31.7,-15.1) |
| Non-Latina, Asian/Pacific Islander/AI/AN/Other^2^ | 22.4 (4.3,43.6) |
| Latina | -0.9 (-12.4,12) |
| Primary payer/health insurance |  |
| Private | Reference |
| Uninsured | -35.9 (-44.5,-26) |
| Medicaid | -36.3 (-44.2,-27.2) |
| Medicare | -20.4 (-27.6,-12.5) |
| Other | -9.3 (-21,4.1) |
| Year of diagnosis, per 1 standard deviation (sd) | 2.3 (-1.3,6.1) |
| Cancer stage |  |
| Early |  |
| Late |  |
| Grade |  |
| Low | Reference |
| High | -55.4 (-58.5,-52) |
| Tumor subtype |  |
| Non-triple negative | Reference |
| Triple negative | -48.8 (-52.7,-44.5) |
| Census-based neighborhood factors, per 1 sd |  |
| Socioeconomic composition | 6.2 (1.4,11.1) |
| AA residential density^3^ | 14.0 (1.5,27.9) |
| AA residential segregation, Gini Index^3^ | 0.9 (-3.6,5.7) |
| AA residential segregation, Isolation Index^3^ | -14.0 (-22.7,-4.4) |
| Latino residential density | -5.2 (-19.6,11.8) |
| Latino residential segregation, Gini Index | -1.6 (-6.4,3.4) |
| Latino residential segregation, Isolation Index | 9.7 (-6.2,28.2) |
| Population density | -3.0 (-7.1,1.4) |
| Primary care physician density | 2.0 (-1.7,5.9) |

^1^ Expressed as % change in survival time (TR-1) x 100%; negative values indicate shorter survival time and positive values indicate longer survival time

^2^ AI = American Indian, AN = Alaska Native

^3^ AA = African American

Supporting Table 4. Hazard ratios of BrCa mortality by physical disorder, sociodemographic, tumor, and area-level factors from a Cox shared frailty model, NJSCR BrCa cases, 2008-2017^1^

|  | HR (95% CI) |
| --- | --- |
| Residential physical disorder, 1 sd |  |
| Among early stage | 1.12 (1.07,1.18) |
| Among late stage | 1.01 (0.95,1.08) |
| Age, 1 yr | 1.02 (1.02,1.02) |
| Race ethnicity |  |
| Non-Latina, White | 1.00 |
| Non-Latina, Black | 1.33 (1.18,1.49) |
| Non-Latina, Asian/Pacific Islander/AI/AN/Other^2^ | 0.81 (0.68,0.96) |
| Latina | 1.01 (0.89,1.15) |
| Primary payer/health insurance |  |
| Private | 1.00 |
| Uninsured | 1.60 (1.38,1.87) |
| Medicaid | 1.61 (1.40,1.85) |
| Medicare | 1.27 (1.15,1.40) |
| Other | 1.11 (0.96,1.28) |
| Year of diagnosis, per 1 standard deviation (sd) | 0.95 (0.91,0.99) |
| Grade |  |
| Low | 1.00 |
| High | 2.34 (2.18,2.52) |
| Tumor subtype |  |
| Non-triple negative | 1.00 |
| Triple negative | 2.05 (1.89,2.23) |
| Census-based neighborhood factors, per 1 sd |  |
| Socioeconomic composition | 0.94 (0.89,0.99) |
| AA residential density^3^ | 0.87 (0.77,0.99) |
| AA residential segregation, Gini Index^3^ | 0.99 (0.94,1.04) |
| AA residential segregation, Isolation Index^3^ | 1.17 (1.04,1.32) |
| Latino residential density | 1.06 (0.89,1.27) |
| Latino residential segregation, Gini Index | 1.02 (0.96,1.08) |
| Latino residential segregation, Isolation Index | 0.91 (0.76,1.07) |
| Population density | 1.03 (0.98,1.08) |
| Primary care physician density | 0.98 (0.94,1.02) |

^1^ Shared frailty for cases nested within census tracts

^2^ AI = American Indian, AN = Alaska Native

^3^ AA = African American

References

Krieger, N. (2001). Theories for social epidemiology in the 21st century: an ecosocial perspective. *International journal of epidemiology, 30*(4), 668-677.

Krieger, N. (2011). *Epidemiology and the people's health: theory and context*. New York: Oxford University Press.

Krieger, N. (2013). History, biology, and health inequities: Emergent embodied phenotypes and the illustrative case of the breast cancer estrogen receptor. *American Journal of Public Health, 103*(1), 22-27.

Krieger, N., Jahn, J. L., & Waterman, P. D. (2017). Jim Crow and estrogen-receptor-negative breast cancer: US-born black and white non-Hispanic women, 1992-2012. *Cancer Causes Control, 28*(1), 49-59. doi:10.1007/s10552-016-0834-2

Saini, G., Ogden, A., McCullough, L. E., Torres, M., Rida, P., & Aneja, R. (2019). Disadvantaged neighborhoods and racial disparity in breast cancer outcomes: the biological link. *Cancer Causes & Control, 30*(7), 677-686. doi:10.1007/s10552-019-01180-4

Williams, D. R., Mohammed, S. A., & Shields, A. E. (2016). Understanding and effectively addressing breast cancer in African American women: Unpacking the social context. *Cancer*.
